# Quality Control Strategy for CRISPR-Cas9-Based Gene Editing Complicated by a Pseudogene

**DOI:** 10.3389/fgene.2019.01297

**Published:** 2020-01-08

**Authors:** Zoé Hanss, Ibrahim Boussaad, Javier Jarazo, Jens C. Schwamborn, Rejko Krüger

**Affiliations:** ^1^Clinical and Experimental Neuroscience, Luxembourg Centre for Systems Biomedicine (LCSB), University of Luxembourg, Belvaux, Luxembourg; ^2^Developmental and Cellular Biology, Luxembourg Centre for Systems Biomedicine (LCSB), University of Luxembourg, Belvaux, Luxembourg; ^3^Parkinson Research Clinic, Centre Hospitalier de Luxembourg (CHL), Luxembourg, Luxembourg; ^4^Transversal Translational Medicine, Luxembourg Institute of Health, Strassen, Luxembourg

**Keywords:** CRISPR-Cas9, GBA, pseudogene, GBAP1, iPSC, Parkinson’s disease

## Abstract

CRISPR-Cas9 mediated gene editing in induced pluripotent stem cells became an efficient tool to investigate biological mechanisms underlying genetic-driven diseases while accounting for the respective genetic background. This technique relies on the targeting of a specific nucleotide sequence present in the gene of interest. Therefore, the gene editing of some genes can be complicated by non-coding pseudogenes presenting a high homology of sequence with their respective genes. Among them, *GBA* is raising special interest because of its implication as the most common genetic risk factor for Parkinson’s disease. In this study, we present an easy-to-use CRISPR-Cas9 gene editing strategy allowing for specific editing of point mutations in a gene without genetic alteration of its pseudogene exemplified by the correction or insertion of the common N370S mutation in *GBA*. A quality control strategy by combined fluorescence and PCR-based screening allows the early identification of correctly edited clones with unambiguous identification of the status of its pseudogene, *GBAP1*. Successful gene editing was confirmed by functional validation. Our work presents the first CRISPR-Cas9 based editing of a point mutation in *GBA* and paves the way for technically demanding gene engineering due to the presence of pseudogenes.

## Introduction

Pseudogenes are DNA sequences with high homology to functional genes. More than 17,000 pseudogenes have been identified in the human genome and up to 10% of them are transcribed (Kovalenko and Patrushev, 2018). Even if pseudogenes are considered as ancient genes that have lost their functions (Portin, 2002), they still participate to the regulation of their parental gene expression at both transcriptional and translational levels (Kovalenko and Patrushev, 2018). Pseudogenes can act as competing endogenous RNA (ceRNA) toward their parental gene and compete for regulatory microRNA (miRNA) (Costa et al., 2012; Kovalenko and Patrushev, 2018). By these mechanisms, the ratio between the amount of gene and pseudogene transcripts modulates the expression of the gene. Consequently of these functionalities, pseudogenes have been implicated in the development of human diseases, in particular in cancer and neurodegenerative disorders (Costa et al., 2012).

Genetic modification of living cells and organisms has taken a major leap in the last decades due to the development of targeted nucleases (Kim, 2016). Whether it is TALEN (Transcription-activator-like effector nuclease), ZFN (Zinc finger nuclease), or CRISPR (Cluster of regularly interspaced palindromic repeats)-Cas9 (CRISPR-associated protein 9), all these gene editing techniques rely on the recognition of specific base-pair sequences to allow the targeting of the gene of interest (Sander and Joung, 2014). Therefore, the presence of highly homologous pseudogenes complicates the specific targeting of their respective gene. This implies two risks: first, the decreased efficiency of the targeting caused by off targets due to highly similar sequence; second, the unintentional modification of the pseudogene sequence leading to unbalanced ratio of gene/pseudogene transcripts.

One well known pseudogene of a disease-associated functional gene is *GBAP1* (Horowitz et al., 1989). The exonic region of *GBAP1* shares 96% of homology with the *GBA* gene (Martínez-Arias et al., 2001). Homozygous mutations in *GBA* cause Gaucher’s disease (GD) (Stirnemann et al., 2017), and recently heterozygous *GBA* mutations have been identified as the most common genetic risk factor for developing Parkinson’s disease (PD) (Sidransky et al., 2009). With 5–15% of PD patients carrying *GBA* mutations (Neumann et al., 2009), studies of *GBA* and its encoded enzyme glucocerebrosidase (GCase) became a high priority in PD research. The two most common mutations, p.N370S and p.L444P, are located in exons 9 and 10 respectively. Unfortunately, the sequence homology between *GBAP1* and *GBA* reaches 98% from intron 8 to the 3’ untranslated region (Zampieri et al., 2017). Consequently, this sequence similarity makes it difficult to specifically sequence *GBA* (Zampieri et al., 2017) and greatly complicates the specific targeting of this region by gene editing. Therefore, to date, most of the *GBA* gene-engineered models on human-derived cells rely on knock-out (KO) of *GBA via* targeting of exons 3 and 4 (Kim et al., 2018; Schöndorf et al., 2018). Nevertheless, these models fail to replicate the genotype observed in GD or *GBA*-PD patients. Only one study performed the correction of point mutations of *GBA* in patient-derived induced pluripotent stem cells (iPSC) *via* ZFN-mediated homologous recombination (Schöndorf et al., 2014). Recently, CRISPR-Cas9 technology emerged as an accessible, reliable, and efficient gene editing method for insertion or correction of point mutations (Arias-Fuenzalida et al., 2017). Therefore we undertook the editing of *GBA* in patient-derived iPSCs by FACS-assisted CRISPR-Cas9 technology (Jarazo et al., 2019).

In this study, we describe a step-by-step strategy that allows successful gene editing of a gene while ensuring the integrity of its pseudogene. We developed a CRISPR-Cas9 based technique allowing the correction and insertion of point mutations in *GBA* without alteration of its pseudogene, *GBAP1*.

## Material and Methods

Methods for preparation of vectors (sgRNA and donors) are described in the supplemental information. The iPSC lines used in this study were reprogrammed from patient-derived fibroblasts using Simplicon RNA Reprogramming Kit (Merck) and the expression of the pluripotency markers *Nanog*, *Oct4*, and *SOX2* was validated by immunocytochemistry (**Figure S1**). iPSCs were maintained on Geltrex (Gibco) coated plates in E8 medium supplemented with 10% mTesR^®^. When cells were platted as single cells or picked, the medium was supplemented with 10 µM of Y-27632 (Rock inhibitor). For PCR-based screening, colonies were lysed in colony-PCR lysis buffer (Papapetrou and Sadelain, 2011) and 3 µl of the lysate was used for PCR amplification. The HumanOmni2.5 Exome-8 DNA Analysis BeadChip was used to evaluate chromosomal aberration (performed by LIFE & BRAIN GmbH, Bonn). The GCase activity assay was performed as described (Mazzulli et al., 2011) and fluorescence measurement was performed on an Infinite^®^ M200 PRO (Tecan). GCase protein level was evaluated by Western blotting. Comprehensive information on the experimental procedures is described in the Supplemental Information.

## Results

### Generation of Donor Plasmids and sgRNA to Gene-Edit the N370S Mutation in *GBA*

**Figure 1:**
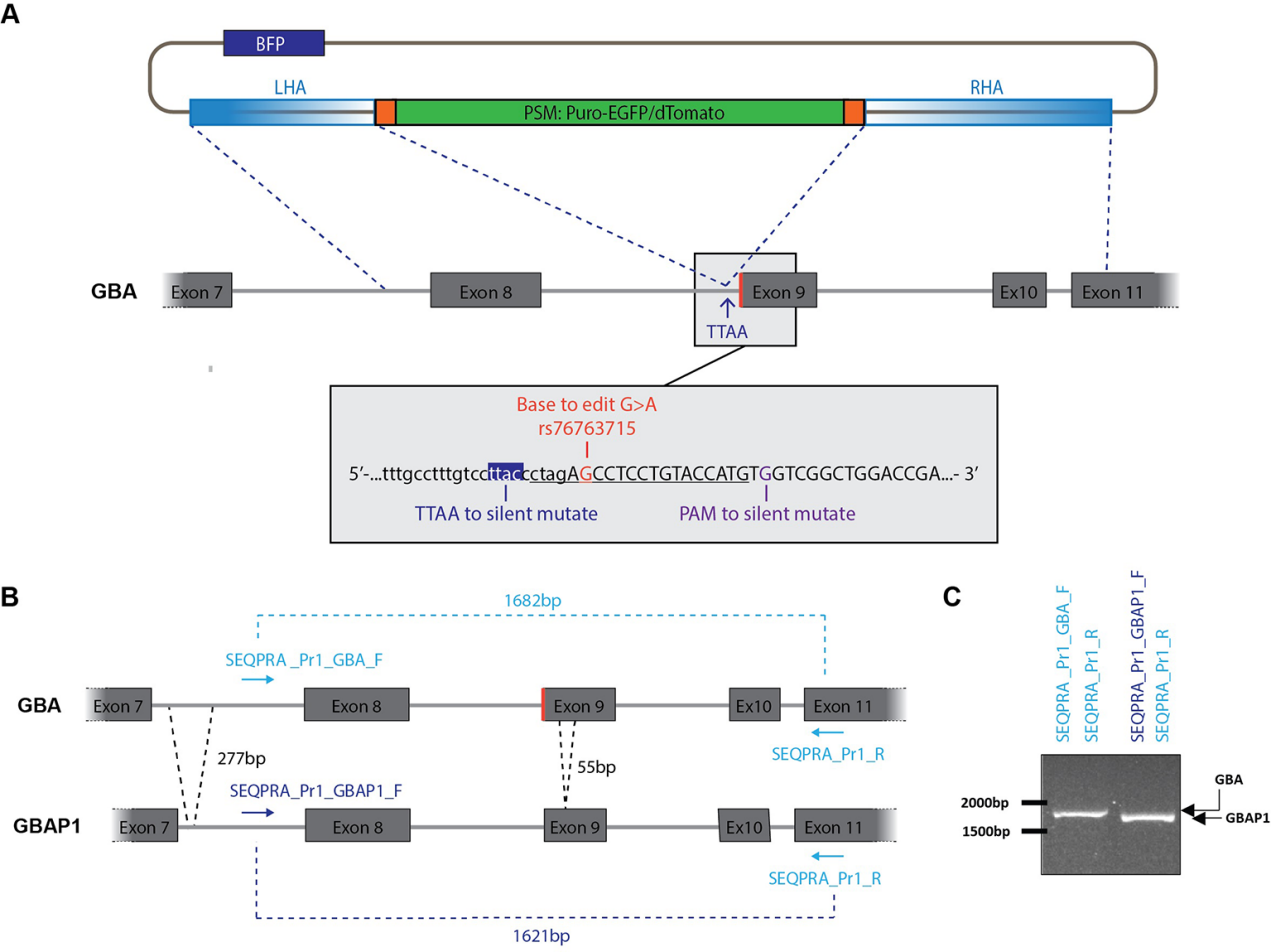
Construction of donor vector for homology directed repair (HDR). **(A)** The left homology arm (LHA) and the right homology arm (RHA) amplified from genomic DNA are flanking the positive selection module (PSM) which encodes a puromycin selection cassette and either EGFP or dTomato. A BFP, located in the backbone of the vector, allow to identify random integration events. Grey box: Genomic DNA sequence surrounding the mutation rs76763715. The sequence underlined is recognized by the sgRNA and followed by the PAM motif. Two nucleotides will be mutated within the RHA: C > A to generate a TTAA site, G > C in the PAM motif to avoid subsequent recognition after editing. Exons are in uppercase and introns in lowercase. **(B)** Strategy to amplify specifically *GBA* over *GBAP1* for construction of the donor vector. *GBAP1* presents two deletions in its sequence compared to *GBA*: a 277 bp deletion in intron 8 and a 55 bp deletion in exon 9. **(C)** Validation of the specificity of selected SEQPRA primers. Visualization on an agarose gel allows to discriminate the amplicon of *GBA* from *GBAP1* after PCR amplification with the selected primers.

Exon 9 of *GBA* is a hot spot of pathological mutations (Dandana et al., 2016). To illustrate the possibility of specifically targeting this exon, we selected the mutation rs76763715 (transition c.1226A > G) resulting in an amino-acid exchange (p.N370S) in GCase. This mutation was either corrected or inserted at the heterozygous state in patient-derived iPSCs in order to model its implication in PD. The gene editing strategy is based on the protocol published earlier this year by Jarazo and colleagues (Jarazo et al., 2019). The process makes use of two donor vectors for homology directed repair allowing deterministic identification of the gene editing status by a fluorescence-based approach (**Figure 1A**). One vector encodes a red fluorescent protein (dTomato), while the other one encodes a green fluorescent protein (EGFP). Therefore, biallelic targeting can be determined by screening of double-positive clones (dTomato+/EGFP+). In the backbone of each donor, a negative selection module (NSM) containing a blue fluorescent protein (BFP) provides identification of random-integration events (**Figure 1A** and **Figure S2H**). The positive selection module (PSM) composed of dTomato or EGFP and of a puromycin selection cassette is flanked by the piggyBac inverted terminal repeats (ITRs) for removing of the cassette after selection of correctly edited clones by transfection with the excision-only transposase (**Figure 1A** and **Figure S2H**). The removal results in the formation of a TTAA motif in the host genome (Yusa et al., 2011). Therefore, the PSM has to be inserted within an existing TTAA sequence of the DNA to be edited or, if necessary, a TTAA can be created by silent mutation.

As we wanted to correct or insert the N370S mutation in *GBA* at the heterozygous state, three donor vectors were designed. One carried the mutation in the homology arms associated with dTomato. The two others carried the wild-type (WT) codon associated either with dTomato or EGFP. To increase our chance of successful gene editing, both biallelic and monoallelic repair events were selected. Indeed, monoallelic repair can also correspond to a correct heterozygous editing of *GBA*, however both alleles have to be sequenced to ensure that a non-homologous end joining event (NHEJ) didn’t occur in the allele without the PSM. Each step of the editing process, from design to final screening, took into account the presence of the highly homologous pseudogene *GBAP1*, located 16 kb downstream of *GBA*, and therefore ensure specific editing of *GBA*.

The mutation rs76763715 is located within the first codon of exon 9 of *GBA* and results in the transition AAC (WT codon) to AGC (**Figure 1A**). We selected this codon as the center of the foreseen donor construct surrounded with 2 kb upstream and downstream. After analyzing this region for repetitive elements with Repeat Masker tool (http://www.repeatmasker.org/), we noticed three short repetitive elements and one ALU sequence within the selected DNA region. To limit the risk of homology with any other DNA region containing these repetitive elements, we reduced the size of our donor template until reaching a satisfying balance between specificity and sufficient length. To ensure specific amplification of *GBA* over its pseudogene, we took advantage of the few sequence differences existing between *GBA* and *GBAP1* in the region of interest. Indeed, *GBAP1* contains a 55 bp deletion in exon 9, a 277 bp deletion, and several base pair differences in the intron 7 compared to *GBA* (**Figure 1B**). The selected forward primer (SEQPRA_Pr1_GBA_F) has five mismatches with the *GBAP1* sequence to ensure the specific amplification of *GBA*. We controlled for specificity of this amplification by using a forward primer matching the *GBAP1* sequence (SEQPRA_Pr1_GBAP1_F) which generate a shorter amplicon due to the deletions in *GBAP1* (1621 bp, **Figures 1B**, **C**). The amplicons resulting from amplification of *GBA* and *GBAP1* with their respective SEQPRA primers have 95% homology. The region of interest in *GBA* was amplified from patient’s DNA harboring the heterozygous N370S mutation and sub-cloned into a TOPO vector resulting into two different vectors: one carrying the mutation and the other the WT codon.

From both of these vectors, we amplified the homology arms to be cloned into the final donor vector around the PSM (**Figure 1A** and **Figure S2**). Briefly, the homology arms were amplified from the TOPO vectors described above with overhangs matching the donor vector scaffold after HpaI enzymatic digestion (**Figure S2A**, **Table S1**) (Jarazo et al., 2019). The right homology arm (RHA) was generated in two steps allowing introduction of a TTAA motif and mutation of the PAM sequence. We identified a TTAC only 6 bp downstream of the codon of interest and located in the intronic region preceding exon 9 (**Figure 1A**). Therefore, we introduced a TTAA motif without interfering with amino-acid sequence (**Figure S2G**). The PAM sequence was modified by silent mutation G > C (**Figure S2G**). The assembly of the arms into the donor vector scaffold was performed as described (**Figures S2H, I**) (Jarazo et al., 2019). At the end of this process, three donor vectors were obtained: one carrying the mutation associated with dTomato (dTomato_AGC), one carrying the WT codon associated with dTomato (dTomato_AAC) and one carrying the WT codon associated with EGFP (EGFP_AAC).

The high sequence similarity between *GBA* and *GBAP1* didn’t allow for generation of a single-guide RNA (sgRNA) specific to the *GBA* sequence. Still, we took advantage of the presence of the mutation to create one mismatch between sgRNA and *GBAP1* when correcting the mutation. Therefore, we selected two sgRNA, targeting either the strand harboring the mutation (5’-CTAGA**G**CCTCCTGTACCATG -3’) or the WT strand (5’- CTAGA**A**CCTCCTGTACCATG -3’) (**Figure 1A**, underlined). Both were cloned into the Cas9-containing pX330 vector (Addgene 42230) following the protocol from Ran and colleagues (Ran et al., 2013). Ultimately, we obtained two sgRNA vectors: one for correction of the mutation (sgRNA_for_Mut) and one for the insertion of the mutation into the WT allele (sgRNA_for_WT).

### Editing of the *GBA* N370S Mutation in iPSCs and Selection of Correctly Edited Clones by Combined Screening Strategy

For correction of the heterozygous N370S mutation, *GBA*-mutant iPSCs (L1) were co-transfected with the sgRNA-Cas vector sgRNA_for_Mut and with both donor vectors carrying the WT codon, associated either with dTomato or EGFP (dTomato_AAC and EGFP_AAC). For insertion of the heterozygous N370S mutation, *GBA*-WT iPSCs (L2) were co-transfected with the sgRNA-Cas vector sgRNA_for_WT, with the donor vector carrying the mutation associated with dTomato (dTomato_AGC), and with the donor vector carrying the WT codon associated with EGFP (EGFP_AAC).

**Figure 2:**
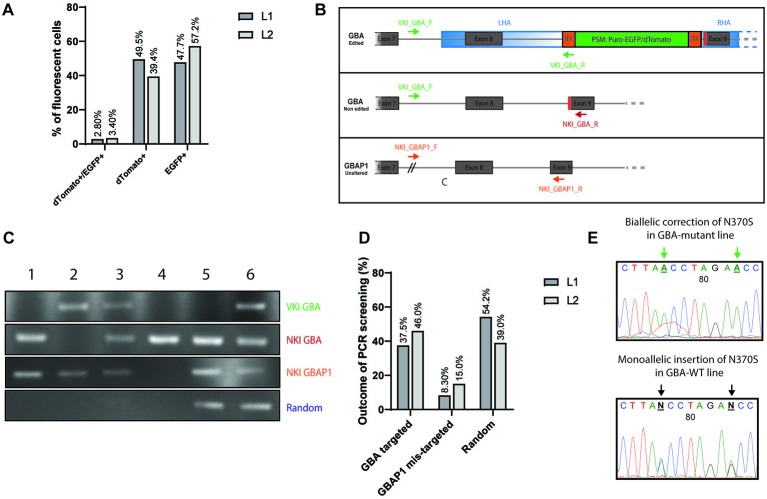
Fluorescent and PCR-based screening of iPSC clones. **(A)** Graphic representation of the outcome of the fluorescent-based screening. Results are expressed in % of all fluorescent colonies (dTomato+, EGFP+, and double-positive). Dark grey: line L1 to correct (739 colonies in total). Light grey: line L2 to insert (522 colonies in total). **(B)** Visualization of the PCR-based screening strategy for the validation of the knock-in (KI). Three editing status need to be screened: correct editing of *GBA* (upper panel, VKI_GBA primers), no editing of *GBA* (middle panel, NKI_GBA primers), no targeting of *GBAP1* (lower panel, NKI_GBAP1). **(C)** Example panel of the different editing status. First lane: Amplification with VKI_GBA primers; second lane: Amplification with NKI_GBA primers; third lane: Amplification with NKI_GBAP1 primers; fourth lane: Amplification with VKI_LHA primers. Clone 1: unedited; Clone 2: correct biallelic targeting of *GBA*; Clone 3: correct monoallelic targeting of *GBA*; Clone 4: mis-targeting of *GBAP1*; Clone 5: random integration LHA; Clone 6: monoallelic targeting of *GBA* and random integration of LHA. **(D)** Graphic representation of the outcome of the PCR-based screening. Results are expressed in % of all clones entering the PCR-based screening (24 clones for L1; 13 clones for L2). Random accounts for clones with random integration only or associated with correct targeting of *GBA*. Dark grey: line L1 to correct. Light grey: line L2 to insert. **(E)** DNA chromatograms of *GBA* sequence after gene editing. Change in the base to edit can be observed as well as the appearance of the TTAA motif after excision of the PSM. Here are examples of biallelic correction in L1 (homozygous for TTAA) and monoallelic insertion for L2 (heterozygous for TTAA).

After transfection, iPSCs were allowed to form colonies and were subsequently subjected to selection *via* puromycin treatment (0.25-0.75 ng/µl, 3 days). After 5–7 days, the remaining puromycin-resistant colonies were processed by fluorescent-based screening (**Figures S3A–D**). The screening of BFP allowed to identify the colonies carrying random-integration events as the BFP present in the backbone of the donor vector should not be integrated in the case of correct homology template repair (**Figures S3C, D**) (Jarazo et al., 2019). Among all puromycin-resistant colonies that were expressing EGFP and/or dTomato, 3% were double-positive dTomato+/EGFP+ (**Figure 2A** and **Figure S3A**). The other colonies were single-positive dTomato+ or EGFP+ (**Figure 2A** and **Figure S3B**), therefore corresponding to monoallelic homologous repair events. Colonies with ambiguous BFP status were rather picked than discarded, and the blue fluorescence was again evaluated after expansion of the picked clones. For the correction of the N370S in L1, 739 independent colonies were puromycin-resistant and dTomato+ and/or EGFP+. We manually picked 48 monoclonal colonies that were double positive (dTomato+/EGFP+) or single positive (dTomato+ or EGFP+) into new wells as both situations can correspond to heterozygous correction of the mutant allele. Among them, 24 passed the second BFP-fluorescence screening and were selected for further PCR-based screening. For the insertion of the N370S mutation in L2, 522 independent colonies were puromycin-resistant and dTomato+ and/or EGFP+. EGFP single-positive clones were left out as it corresponds to insertion of the WT codon on a WT background. We picked 24 double positive or dTomato+ monoclonal colonies and among them 13 passed the second BFP-fluorescence screening and were selected for PCR-based screening. If after manual picking a clone was presenting a mixed population of cells (**Figure S3D**), a first step of sorting BFP− cells was carried out to obtain a homogenous population (**Figure S4A**).

A PCR-based screening was then conducted to specify the editing status of each independent clone. Indeed, the fluorescence-based screening alone is not sufficient to identify whether *GBA* and/or *GBAP1* was targeted and whether some random-integration events excluding the BFP occurred. To identify the correct editing of *GBA* (validation of knock-in, VKI), we used a forward primer binding outside of the homology arm (VKI_Pr1_GBA_F) associated with a reverse primer binding in the ITR of the PSM (VKI_Pr1_R) (**Figure 2B**, upper panel). The forward primer was designed to bind in the 277 bp region of *GBA* which is deleted in *GBAP1* (**Figure 1B**), therefore allowing a specific amplification of *GBA*. An example of the different outcomes of the PCR screening is displayed in **Figure 2C**. Here we can see that the clones 2, 3, and 6 were correctly targeted in *GBA* (upper lane). To identify if both *GBA* alleles were targeted or not, we used the same forward primer (VKI_GBA_F) but a reverse primer binding in the genomic region after the PSM (NKI_GBA_R) (**Figure 2B**, middle panel). This primer set amplifies a 952 bp sequence only in iPSCs carrying an untargeted *GBA* allele (no knock-in—NKI). We can conclude that both alleles of clone 2 were targeted as no amplification with the second primer pair occurred, while only one of the alleles of clones 3 and 6 were targeted (**Figure 2C**, second lane). To ensure that *GBAP1* was not mis-targeted, we amplified specifically *GBAP1* with a forward primer binding at the junction of the 277 bp deletion (NKI_GBAP1_F) and a reverse primer binding in the 55 bp deletion of exon 9 (NKI_GBAP1_R) (**Figure 2B**, lower panel). If the pseudogene was altered, no amplification would occur with this primer pair, as in clone 4 (**Figure 2C**, third lane), and the clone was discarded. Finally, to identify if random integration events excluding the BFP occurred, we used two primer sets (Jarazo et al., 2019). We identified clones harboring random integration of the vector backbone like clone 5 (**Figure 2C**, fourth lane). Some clones presented a correct targeting of *GBA* and, additionally, random integration like clone 6 (**Figure 2C**, fourth lane).

Clones harboring random integration of the backbone or unintentional targeting of *GBAP1* were discarded (**Figure 2C**, Clones 4, 5, and 6). Clones identified as dTomato+/EGFP+ at fluorescence-screening and exhibiting a biallelic repair of *GBA* were kept and expanded (**Figure 2C**, Clone 2). Clones identified as dTomato+ or EGFP+ at fluorescence-screening and presenting a monoallelic repair of *GBA* were kept and expanded (**Figure 2C**, Clone 3). In case of monoallelic repair of cell lines carrying heterozygous N370S mutation, sequencing of the region of interest was performed to ensure that the mutant allele, and not the WT, was edited.

For the correction of the N370S mutation in the *GBA*-mutant L1 line, 37.5% of the PCR-screened colonies were correctly targeted on *GBA* without alteration of *GBAP1* and without random integration (**Figure 2D**). To note, 8.30% of the screened clones were mis-targeted on *GBAP1* and 54.2% showed random integration of the targeting vector. For the insertion of the mutation in the *GBA*-WT L2 line, 46.0% of the PCR-screened colonies were correctly targeted on *GBA* without alteration of *GBAP1* and without random integration (**Figure 2D**), while 15.0% were mis-targeted on *GBAP1* and 39.0% were harboring random integration of the vector backbone.

Each independent clone was expanded and purified by FACS in order to obtain a homogenous population of fluorescent cells. iPSCs were sorted by gating according to their fluorescence status, *i.e.* double-positive dTomato+/EGFP+ or single-positive for one color (**Figure S4B, D**). A first gate on BFP− cells was used as a safety measurement to eliminate any risk of sorting mixed populations due to contamination of a BFP− colony by neighboring BFP+ cells during the single-colony manual picking step (**Figure S4A, B**). After two to three sortings, 100% of the iPSCs within each selected clone were of the expected fluorescence status (**Figure S4C–F**). At this stage, removal of the PSM was pursued with the transposase enzyme as described (Jarazo et al., 2019) (**Figure S5A**). After transposase treatment, further sorting allowed to obtain uncolored and independent gene-edited iPSC clones (**Figures S5B–E**). A confirmation of the status of *GBA* was performed *via* sequencing (**Table S1**). All selected clones demonstrated the correction or insertion of the heterozygous mutation rs76763715 (p.N370S) (**Figure 2E**). A systematic sequencing of the pseudogene region corresponding to exon 9 was also conducted to confirm that no alteration was caused to *GBAP1* during the gene editing process (**Figure S6**).

All clones were karyotyped and do not present any karyotypic alterations but small duplication of chromosomal region 20q11.21 well known as adaptation of culture condition of iPSCs (Lund et al., 2012; Martins-Taylor and Xu, 2012) (**Figures S7** and **S8**).

### Functional Validation of Successful *GBA* Gene Editing

**Figure 3:**
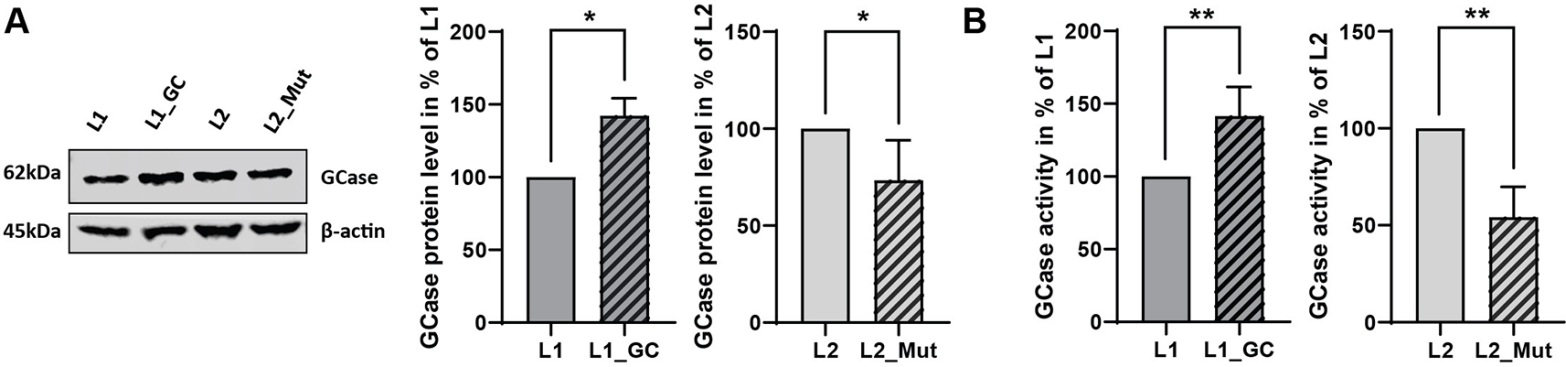
Functional validation of gene-editing of *GBA*. **(A)** Representative immunoblot and densitometry quantification of GCase protein levels in iPSCs expressed in % of non-edited line (mean ± SD, n = 5, two-tailed paired t-test *p < 0.05). **(B)** GCase activity in iPSCs expressed in % of non-edited line (mean ± SEM, n = 5, two-tailed paired t-test **p < 0.005).

To validate the effect of the correction or insertion of the *GBA* N370S in *GBA*-mutant (L1) or *GBA*-WT (L2) derived iPSCs respectively, we explored the protein levels and activity of the encoded enzyme, GCase. The heterozygous N370S mutation in *GBA* is usually characterized by a modest reduction in the protein level and by a decrease of 30 to 50% of GCase activity (Schöndorf et al., 2014; Fernandes et al., 2016; García-Sanz et al., 2017). The correction of the *GBA* N370S mutation in the patient-derived line (L1_GC) induced an increase in the protein level (+42%, P = 0.0255, **Figure 3A**) accompanied by a rise in GCase enzymatic activity (+41.5%, P = 0.0038, **Figure 3B**), therefore rescuing the disease phenotype. On the other hand, the insertion of the mutation in *GBA*-WT line (L2_Mut) led to a decrease in protein level (−27%, P = 0.0449, **Figure 3A**) and to a reduction of GCase activity (−46%, P = 0.028, **Figure 3B**), hence recapitulating the phenotype observed in patient-derived cells harboring a heterozygous *GBA* N370S mutation.

## Discussion

The study of *GBA* is gaining interest since it has been identified as the most common genetic risk factor for PD (Sidransky et al., 2009). To have a better understanding of the cellular mechanisms underlying the pathophysiology, patient-derived cellular models are of great help. Nevertheless, comparison of healthy donors to patient-derived cells does not control for the influence of different genetic backgrounds. Schöndorf and colleagues, provided the first phenotypic readouts on isogenic pairs of iPSC-derived neurons from patients carrying point mutations in *GBA* (Schöndorf et al., 2014). This gene-correction was performed with ZFN which is time-consuming and requires a high technical expertise in order to engineer and select the efficient and non-toxic zinc finger protein to use for editing. Also, to select for corrected clones, a neomycin cassette was introduced in intron 9 of *GBA* and couldn’t be removed after gene-editing. A downstream effect due to the presence of this cassette on intronic regulatory elements cannot be excluded (Patrushev and Kovalenko, 2014). Gene editing of *GBA* was not yet available with a more accessible and simpler technique allowing the integration of the desired modifications only. With this goal in mind, we undertook the targeting of *GBA* by CRISPR-Cas9 FACS-assisted gene editing.

Several studies undertook the gene editing of genes presenting known pseudogenes. Nevertheless, the risks and consequences of an alteration of the pseudogenes were underrated. For example, *PTEN*, a gene implicated in cancer, has been edited by several teams (Wang et al., 2015; Hill et al., 2017), without final sequencing of its pseudogene *PTENP1* even though *PTENP1* is acting as a ceRNA of *PTEN* (Lai et al., 2019). Kawamura and colleagues were confronted with issues related to pseudogene homology when undertaking the CRISPR-Cas9 KO of *NANOG* which has 10 pseudogenes (Kawamura et al., 2015). They could design specific sgRNA for *NANOG* but observed off target effects with deletions of few base-pairs in several pseudogenes. And the alteration in the pseudogenes were identified only at the end of the editing process which represent a loss of time. In the case of *GBA*, Straniero et al. discovered in 2017 that *GBAP1* could act as ceRNA regulating the expression of *GBA via* miRNA (Straniero et al., 2017) and, more recently, a genetic variation of *GBAP1* was associated with gastric cancer risk with over-expression of *GBAP1* promoting cell proliferation, invasion, and metastasis (Ma et al., 2019). Consequently, the unintentional generation of a mutation in *GBAP1* modifying its expression level could have a dramatic effect on the downstream regulation of *GBA* expression and lead to pathological alterations.

From these studies, we identified key steps for successful editing of a gene with a known pseudogene and our work allows to tackle some of these problems: (i) the necessity of proper design of the vectors with specific sgRNA targeting the gene of interest, and the construction of a donor vector encoding the gene sequence and not its pseudogene, (ii) the ability to differentiate early the targeting of the gene from the pseudogene with a discriminating screening strategy, and (iii) the validation of the status of the gene and of its pseudogene by sequencing as a last step. First, for the design of the donor vector, the primer pair used to amplify the homology arms has to ensure the exclusive amplification of the gene without targeting the pseudogene. Indeed, the editing of a gene with a donor vector built from its pseudogene would introduce some sequence modifications that can be pathological. For example, the amplification and subsequent correction of *GBA via* a donor vector constructed from *GBAP1* sequence would introduce the 55 bp deletion in the exon 9 in *GBA* which is a known pathogenic deletion of the gene (Finckh et al., 1998). Here, we could ensure the correct amplification of *GBA* over *GBAP1* by designing a forward primer presenting five mismatches with *GBAP1* (**Figure 1C**). The design of sgRNA targeting specifically the gene over the pseudogene would be helpful to avoid pseudogene mis-targeting. Unfortunately, in our case, the closest sequence differences between the *GBA* and *GBAP1* were located more than 40 bp away from the N370S mutation and it was shown that a distance of more than 30 bp between the double strand break and the base to edit may be associated with reduced gene editing efficiency (Paquet et al., 2016). Moreover, in this area, the only PAM sequence available for silent mutation in the vicinity of a TTAA motif was located on the non-coding strand, which may also reduce the efficiency of the gene editing (Jarazo et al., 2019). Consequently, to balance between efficiency and specificity, a sgRNA binding on the mutation site but potentially targeting *GBAP1* was chosen. However, we could use the sequence differences between the gene and the pseudogene to screen for correctly targeted clones. It is indeed crucial to have a screening strategy enabling the discrimination of the gene from its pseudogene in an early step of the gene editing process. For *GBA*, we made use of the 277 bp deletion in the intron 7 of the pseudogene to specifically evaluate the editing status of *GBA* and of *GBAP1* (**Figures 2B, C**). Moreover, the combined screening strategy *via* fluorescence-directed picking and PCR allowed not only to avoid selection of mis-targeted clones edited on the pseudogene but also to discard randomly integrated clones and to identify biallelic or monoallelic repair of correctly targeted clones. Finally, after removal of the PSM, the last step of sequencing of both the gene and the pseudogene, with discriminating primer pairs, allows to validate the status predicted *via* PCR screening and to select clones edited on the gene without alteration of the pseudogene (**Figure 2D** and **Figure S6**).

Over 300 mutations have been identified in *GBA* (Blandini et al., 2018). Among them, more than 50 are located in the exon 9 (Dandana et al., 2016). Therefore, this gene editing strategy could be used to correct or insert other mutations affecting the exon 9. Both the G377S point mutation (c.1246G > A) and the RecΔ55 mutation (c.1263-1317del; rearrangement between *GBA* and *GBAP1*) could be edited with the same sgRNA as they are less than 30 bp away from the double strand break created with this sgRNA (**Figure S9A**). The donor vectors designed in this study can be used as a template to introduce the minor changes necessary to correct/insert these mutations by site directed mutagenesis. The insertion of the D409H (c.1342G > C) mutation could be conveniently attempted with the sgRNA designed in **Figure S9B** which covers a TTAA site and use the G from the point mutation in its PAM sequence. Moreover, the screening strategy discriminating *GBA* from *GBAP1* developed in this study could also easily be applied to other point mutations found in exon 8 to 11 as the E326K, L444P, or R496H. Furthermore, this gene editing method can also be used to model GD in cellular models as it also allows screening for correction or insertion of the desired *GBA* mutation at the homozygous state.

This study, based on the example of *GBA*, provides a step-by-step strategy allowing the gene editing of a complicated gene due to the presence of a highly homologous pseudogene. It highlights the importance of a well-designed donor vector and of a comprehensive screening strategy when targeting a gene with known pseudogene(s). This first CRISPR-Cas9 based editing of a point mutation in *GBA*, and the description of its application to other mutations, provides a convenient tool that can be widely used for the study of the pathogenic role of *GBA* in both GD and *GBA*-mediated PD models.

## Data Availability Statement

The raw data supporting the conclusions of this article will be made available by the authors, without undue reservation, to any qualified researcher.

## Author Contributions

RK, ZH, and IB conceived and designed the experiments. JS, JJ, and ZH designed the vectors and primers. ZH performed the experiments. ZH drafted the manuscript. All authors contributed to the manuscript revision, read and approved the final version.

## Funding

This work was supported by grants from the Fond National de Recherche within the PEARL programme (FNR/P13/6682797) and the National Centre for Excellence in Research on Parkinson's disease (NCER-PD) programme and by the European Union’s Horizon 2020 research and innovation programme under Grant Agreement No 692320 (WIDESPREAD; CENTRE-PD). JJ is supported by a Pelican award from the Fondation du Pelican de Mie et Pierre Hippert-Faber.

## Conflict of Interest

JJ and JS are inventors in patent PCT/EP2017/051889.

The remaining authors declare that the research was conducted in the absence of any commercial or financial relationships that could be construed as a potential conflict of interest.
