# Supplementary material for: Quality Control Strategy for CRISPR-Cas9-Based Gene Editing Complicated by a Pseudogene
Source: Front Genet. 2020 Jan 8;10:1297. doi: 10.3389/fgene.2019.01297 (PMC6961559; doi:10.3389/fgene.2019.01297)
Supplement: Supplementary file 1 [file DataSheet_1.pdf]

Supplementary information

Supplementary figures:

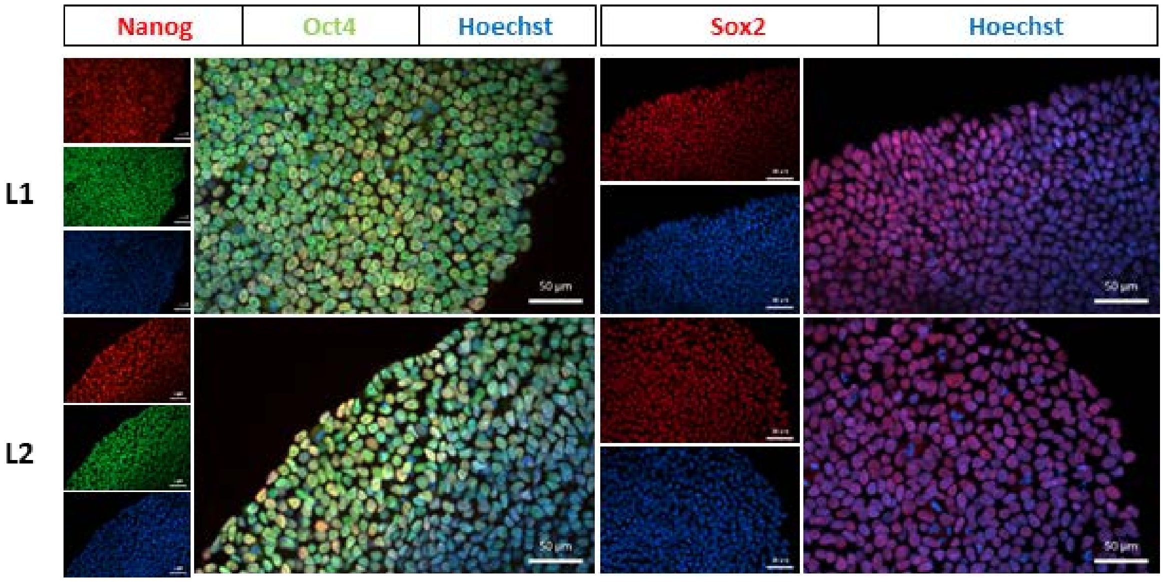

Figure S 1: *Characterization of pluripotency of iPSC lines L1 and L2. Fixed iPSC were incubated with antibodies against pluripotency markers Nanog, Oct4 and Sox2 and detected via fluorescent-conjugated secondary antibodies. Size bar 50µm.*

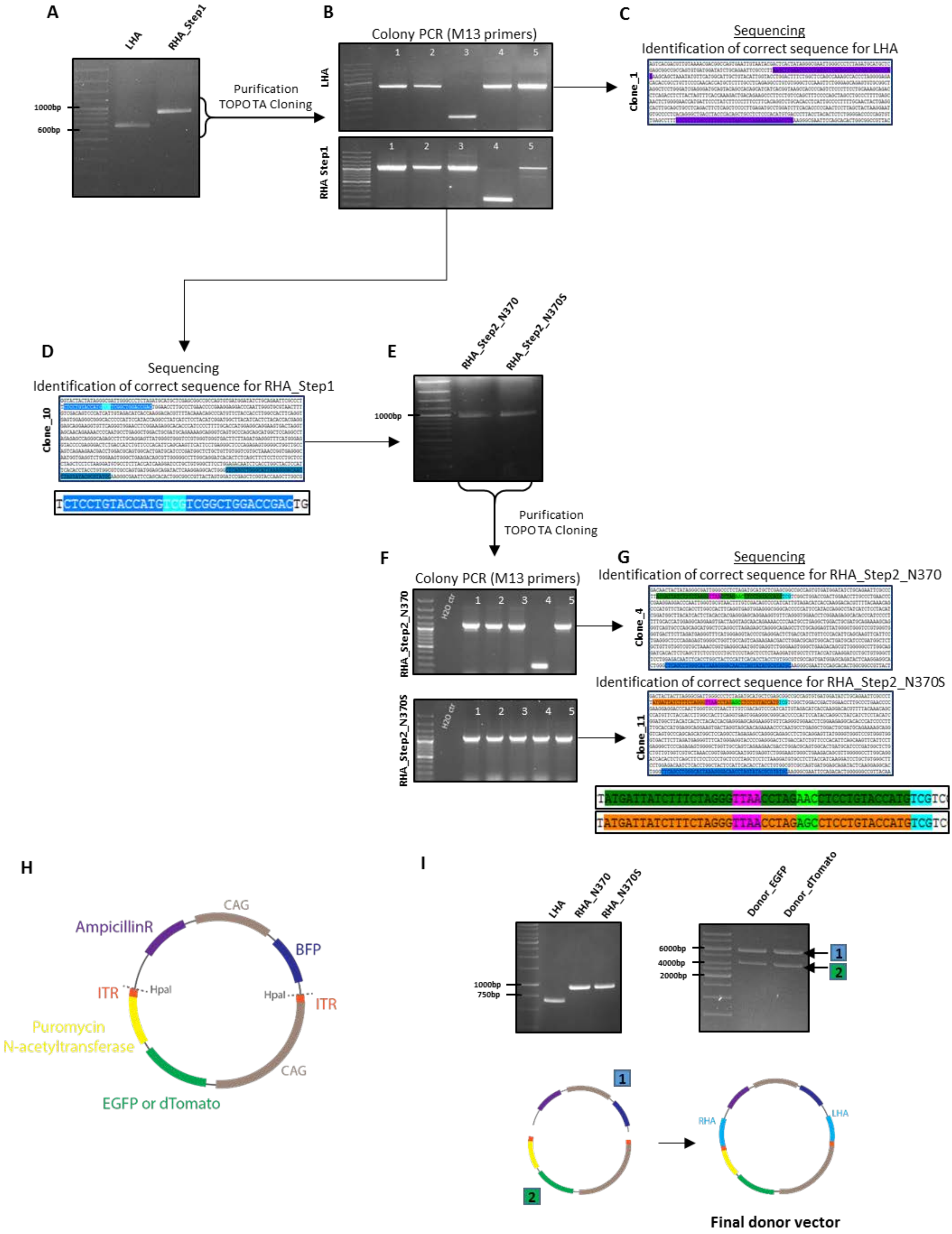

A
LHA
RHA_Step1
1000bp
600bp
Purification
TOPO TA Cloning
B
Colony PCR (M13 primers)
LHA
RHA Step1
1 2 3 4 5
C
Sequencing
Identification of correct sequence for LHA
Clone_1
D
Sequencing
Identification of correct sequence for RHA_Step1
Clone_10
TCTCCTGTACCATGTCGTCGGCTGGACCGACTG
E
RHA_Step2_N370
RHA_Step2_N370S
1000bp
Purification
TOPO TA Cloning
F
Colony PCR (M13 primers)
RHA_Step2_N370
RHA_Step2_N370S
H2O ctr
1 2 3 4 5
G
Sequencing
Identification of correct sequence for RHA_Step2_N370
Clone_4
Identification of correct sequence for RHA_Step2_N370S
Clone_11
TATGATTATCTTTCTAGGGTTAACCTAGAACCTCCTGTACCATGTCGTC
TATGATTATCTTTCTAGGGTTAACCTAGAGCCTCCTGTACCATGTCGTC
H
CAG
AmpicillinR
BFP
ITR
HpaI
HpaI
ITR
Puromycin
N-acetyltransferase
CAG
EGFP or dTomato
I
LHA
RHA_N370
RHA_N370S
1000bp
750bp
Donor_EGFP
Donor_dTomato
6000bp
4000bp
2000bp
1
2
1
2
RHA
LHA
Final donor vector

Figure S 2: Workflow for construction of donor vectors for gene-editing of GBA. (A) Amplification of LHA and RHA from TOPO_HDR. Primers pairs LHA_F with LHA_R and RHA_F_step1 with RHA-R were used. Each unique band was at the expected size, therefore the product of each PCR could be purified and cloned into a TOPO vector. (B) Screening of TOPO_LHA and TOPO_RHA colonies by amplification with M13 primers. Clones presenting a unique band at the respective size of 833 and 1088bp were sequence with the M13 primers. (C) Validation of the sequence of the TOPO_LHA Clone_1. LHA_F and LHA_R binding sequences are highlighted in purple. (D) Validation of the sequence of the TOPO_RHA Clone_10. RHA_F and RHA_R binding sequences are highlighted in blue. Silent-mutation of the PAM sequence is highlighted in light blue. (E) Amplification of RHA_Step2_N370 and RHA_Step2_N370S from TOPO_RHA. Each unique band was at the expected size, therefore the product of each PCR could be purified and cloned into a TOPO vector. (F) Screening of both TOPO_RHA_Step2 colonies by amplification with M13 primers. Clones presenting a unique band at 1088bp were sequence with the M13 primers. (G) Validation of the sequence of the TOPO_RHA_Step2_N370 Clone_4 and TOPO_RHA_Step2_N370S Clone_11. RHA_F_Step2_N370 binding sequence is highlighted in green, RHA_F_Step2_N370S binding sequence is highlighted in orange. Creation of the TTAA motif is highlighted in pink. Respective WT (AAC) or mutant (AGC) codons are highlighted in light green. (H) Representative scheme of pDONOR-tagBFP-PSM-EGFP. Adapted from (Jarazo et al., 2019). *(I) Amplification of LHA and RHA_N370 / RHA_N370S from generated vectors (left pannel). Digestion of EGFP and Tomato empty vectors with HpaI (right pannel). Assembly of the arms with fragments 1 and 2 of the digested donor vector into final donor vector.*

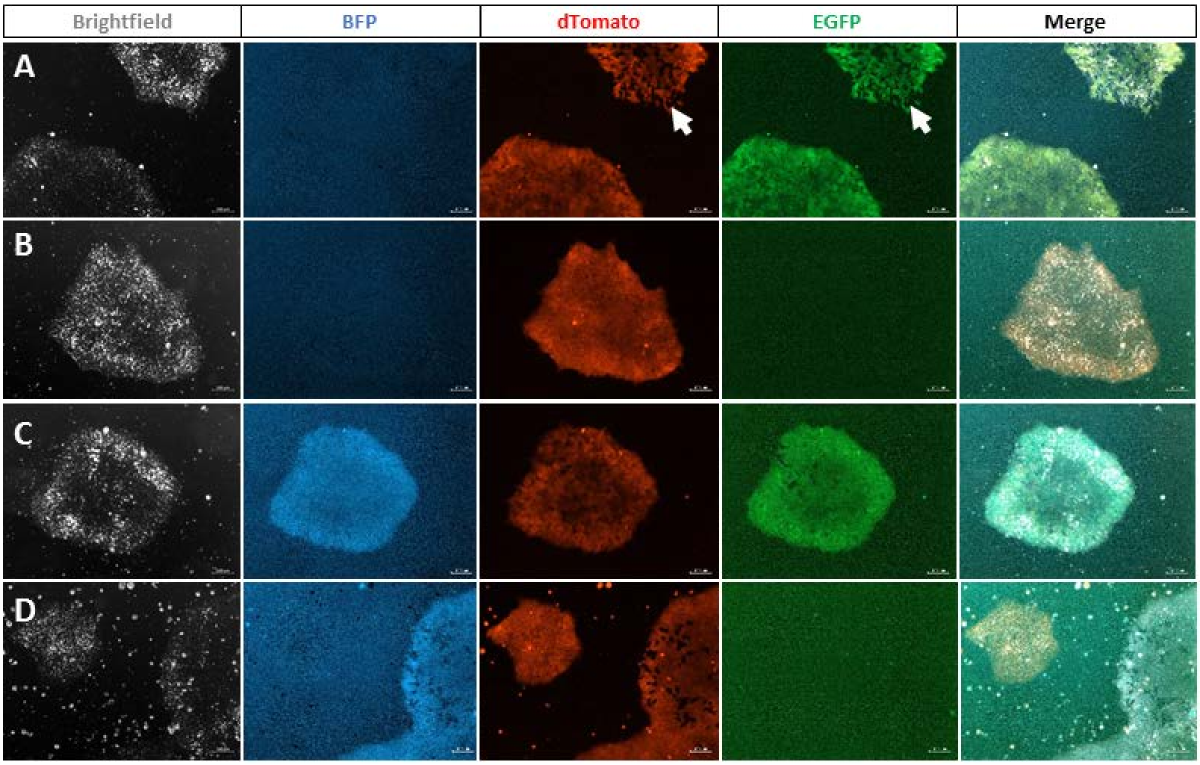

***Figure S 3:** Fluorescent-based screening of puromycin-resistant colonies after picking in 24 well-plate. **(A)** Example of a biallelicly targeted colony (dTomato+/EGFP+) without random integration (BFP-). The white arrow indicates the presence of uncoloured cells. **(B)** Example of a monoallelicly targeted colony (dTomato+/EGFP-) without random integration (BFP-). **(C)** Example of a biallelicly targeted colony (dTomato+/EGFP+) with random integration (BFP+). **(D)** Example of a mixed population presenting monoallelicly targeted colony (dTomato+/EGFP-) with some colonies BFP+ and other BFP-. Size bar 100µm.*

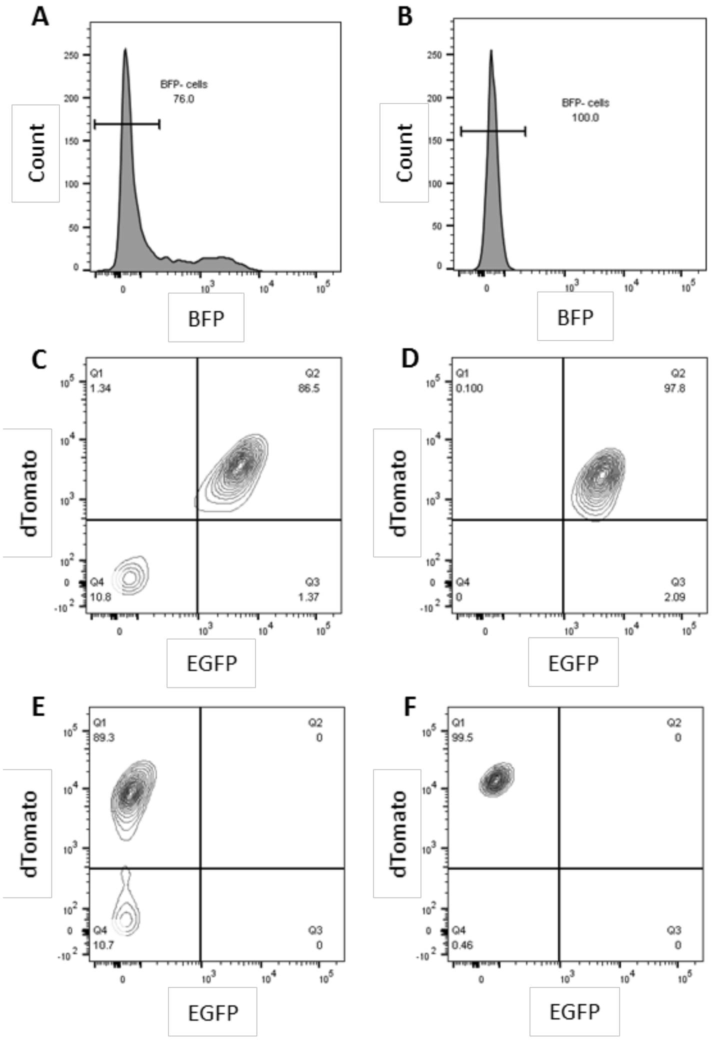

Figure S 4: *Purification of selected clones by FACS.* ***(A)*** *Selection of BFP- cells within a colony presenting a mixed population.* ***(B)*** *Selection of BFP- cells within a colony homogenous BFP- population.* ***(C)*** *First sort of a biallelicly targeted clone. The cells in the upper right area are sorted and expanded (dTomato+/EGFP+).* ***(D)*** *Final sorting of purified biallelicly targeted clone represented in (C).* ***(E)*** *First sort of a monoallelicly targeted clone. The cells in the upper left area are sorted and expanded (dTomato+).* *(****F****) Final sorting of purified monoallelicly targeted clone represented in (E).*

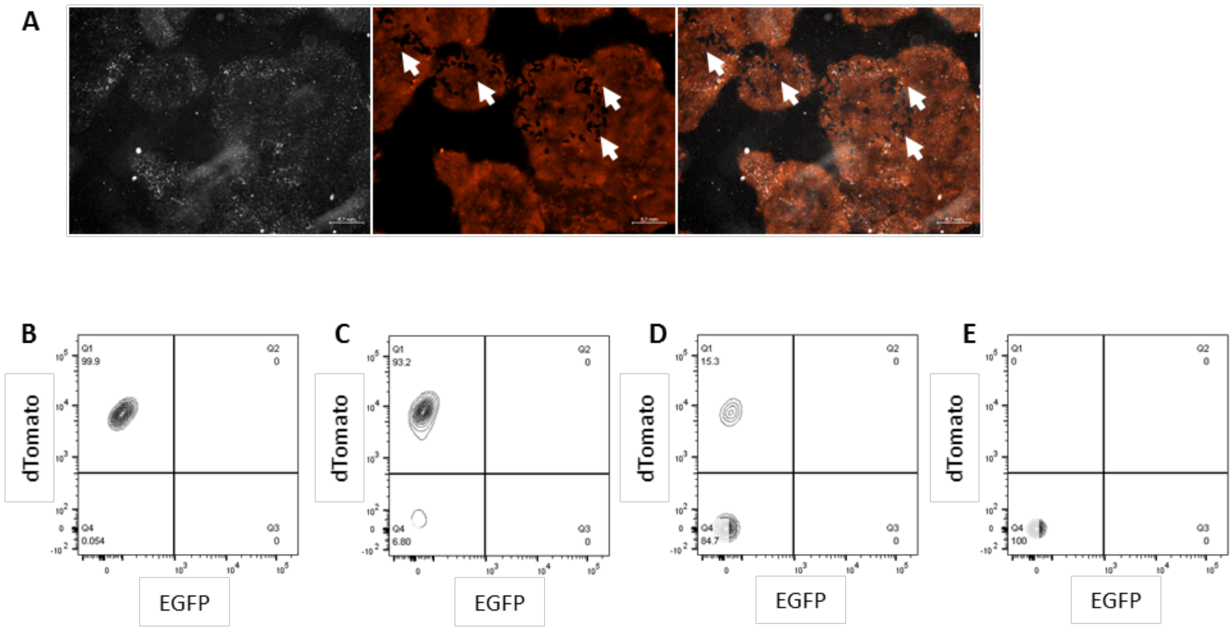

Figure S 5: *Removal of the PSM by excision-only transposase.* ***(A)*** *Loss of the dTomato fluorescence in a monoallelicly targeted clone.* ***(B-E)*** *Sequential sorting of correctly excised cells within a monoallelicly targeted clone:* ***(B)*** *before excision,* ***(C)*** *first sorting after excision. Cells from the lower left panel were sorted in yield mode.* ***(D)*** *Second sorting after excision. Cells from the lower left panel were sorted in purity mode.* ***(E)*** *Third and final sorting after excision. Cells from the lower left panel were sorted in purity mode.*

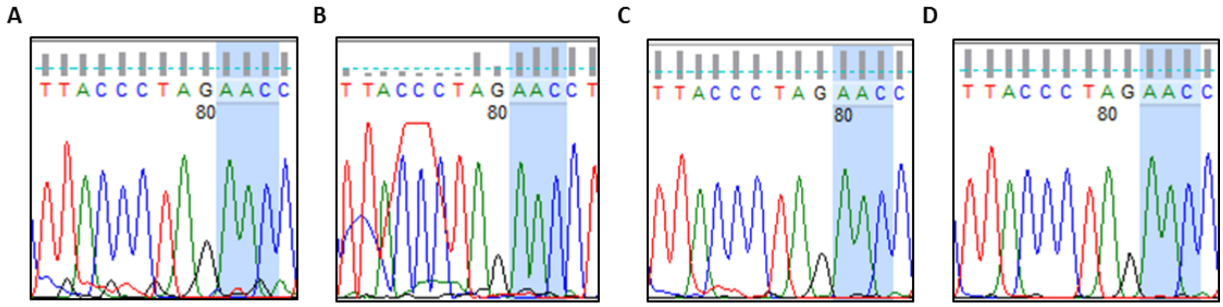

Figure S 6: *Sequencing of GBAP1 after excision of the PSM.* ***(A)*** *L1 (before editing).* ***(B)*** *L1_GC.* ***(C)*** *L2 (before editing).* ***(D)*** *L2_Mut. The sequence of GBAP1 corresponding to the targeted mutation in GBA is highlighted in blue.*

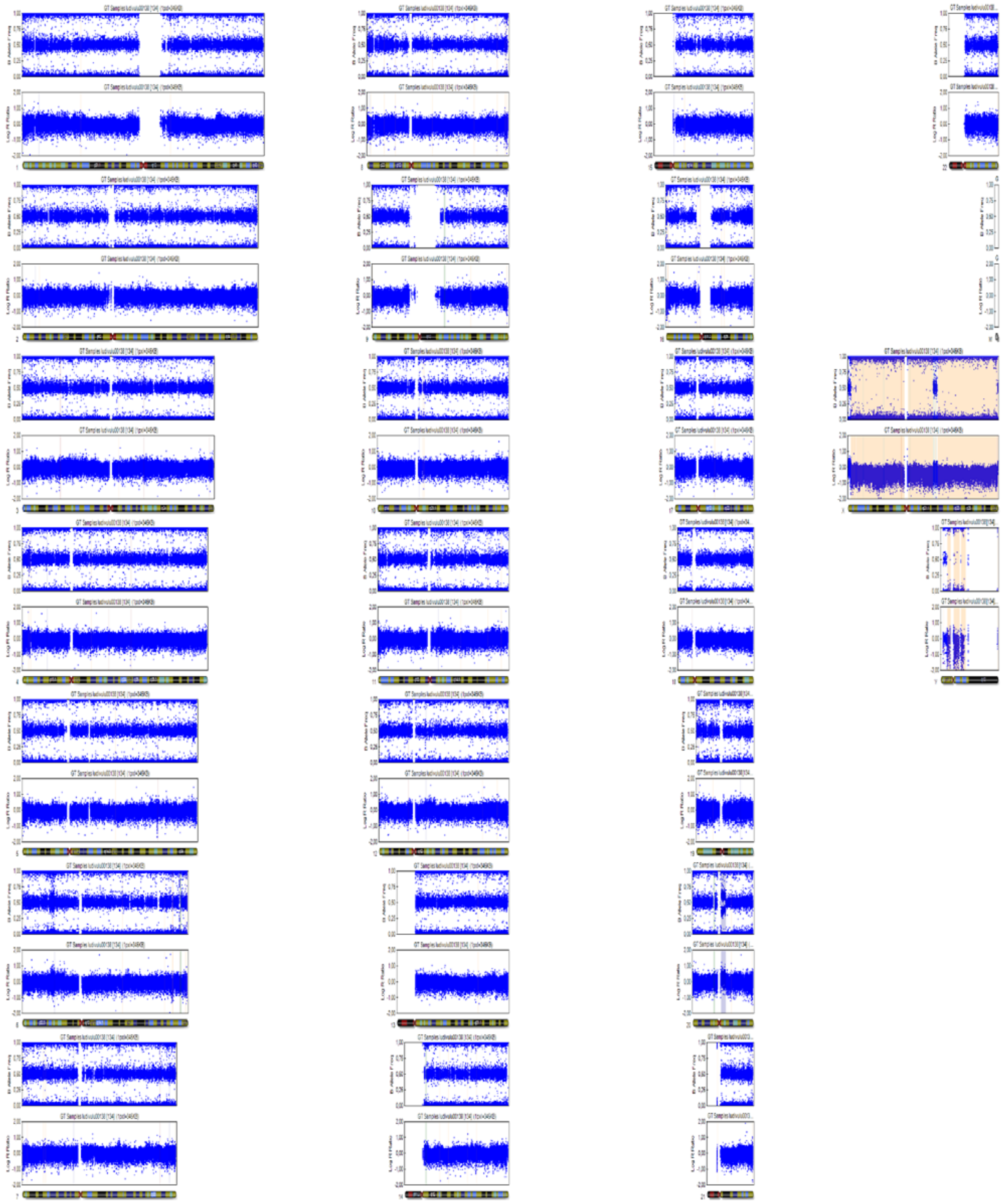

***Figure S 7:** Molecular karyogram of the L1_GC cell line. Single-nucleotide polymorphism assay shows a normal karyotype for a male individual but of small duplication in chromosome 20 due to adaptation to culture conditions.*

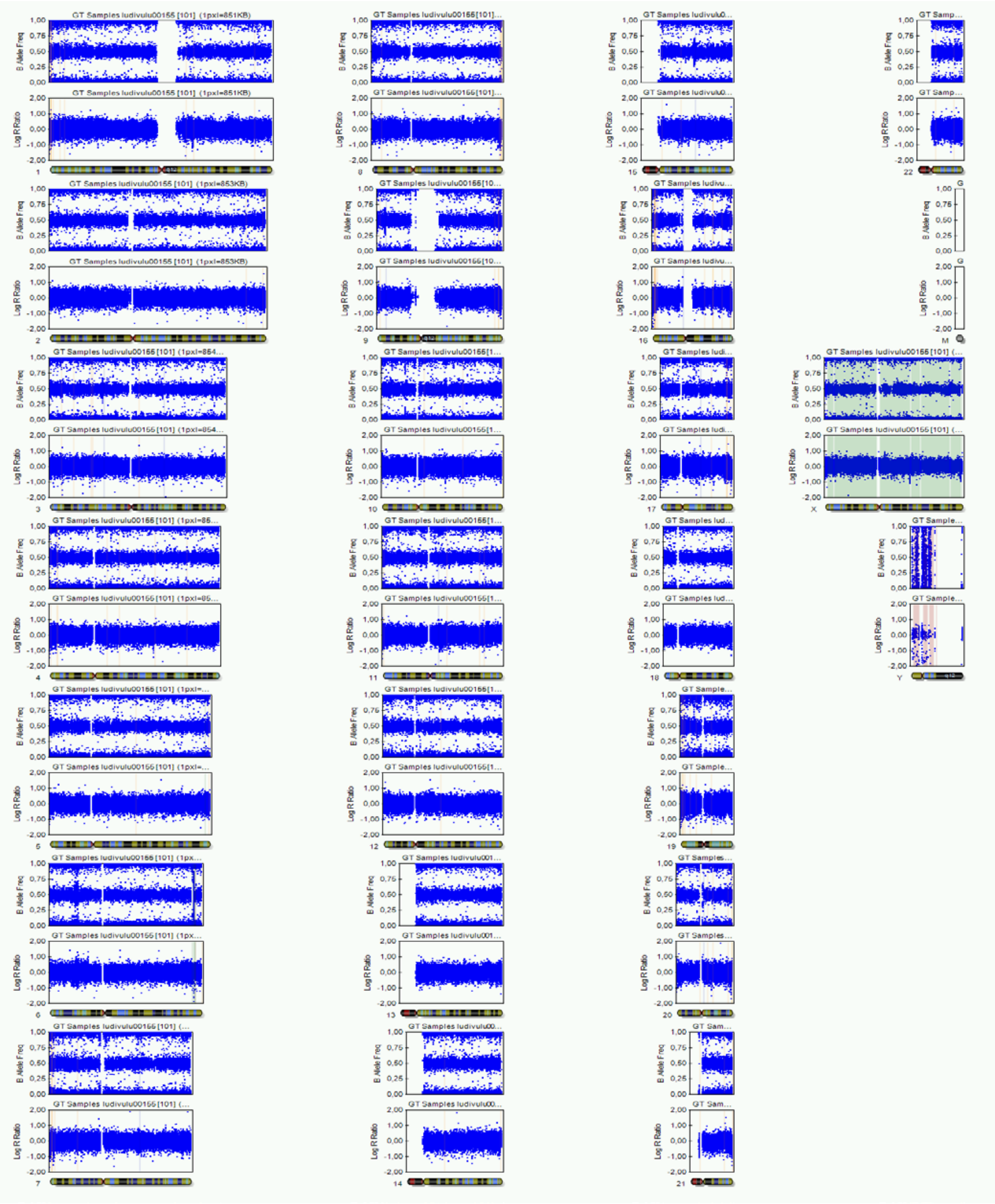

Figure S 8: *Molecular karyogram of the L2_Mut cell line. Single-nucleotide polymorphism assay shows a normal karyotype for a female individual but of a small duplication in chromosome 20 due to adaptation to culture conditions.*

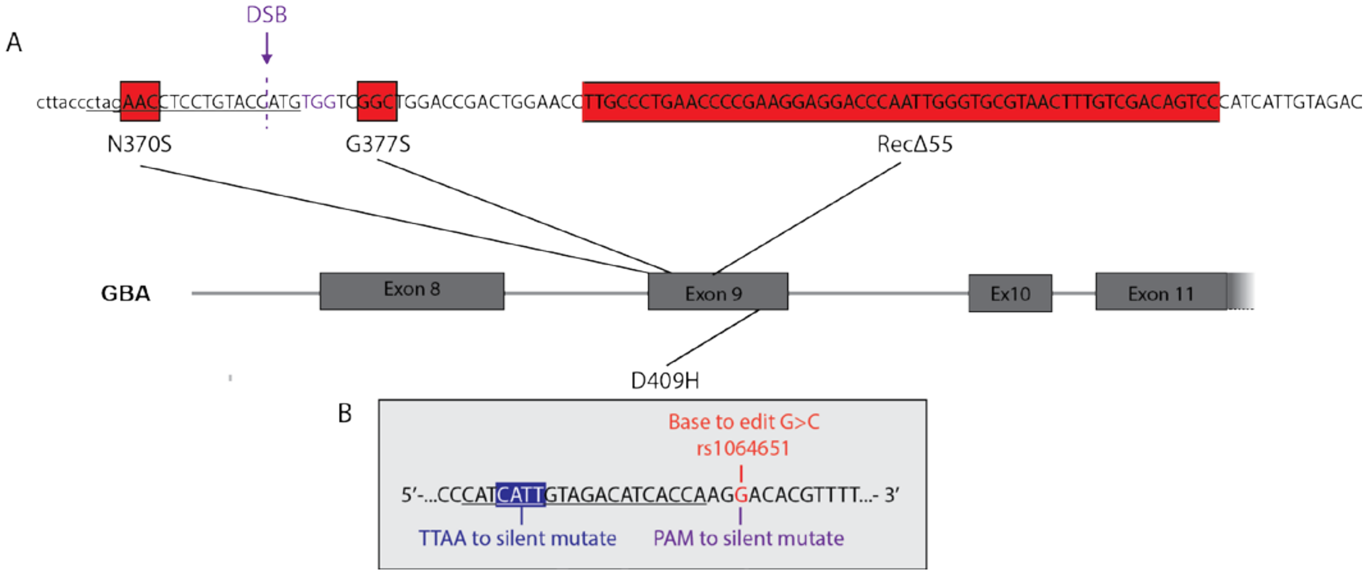

Figure S 9: *Application of the developed gene editing strategy to other GBA mutations.* ***(A)*** *The sgRNA used in this study (underlined) results in a double strand break (DSB) in the vicinity of both the G377S point mutation and the RecΔ55 deletion (highlighted in red).* ***(B)*** *The D409H point mutation could be introduced with the use of another sgRNA (sequence underlined). The creation of the G>C transversion would also mutate the PAM sequence. Here the TTAA motif (highlighted in blue) is on the antisense strand.*

Supplementary material and methods:

DNA extraction

For DNA extraction, iPSC were harvested with Accutase (Sigma-Aldrich) and pelleted by centrifugation at 300g for 3 minutes. The Dneasy Blood and Tissue kit (Qiagen) was used according to the recommendations. Quantity and purity of DNA was assessed using the ratios 260/280nm and 260/230nm on a Spectrophotometer NanoDrop© 2000C (Thermo Scientific).

PCR Amplification for construction of donor vector:

Purified genomic or plasmid DNA was amplified using the GXL polymerase (Takara) as recommended by the manufacturer. 10ng of purified DNA was used for amplification.

TOPO Cloning:

PCR products were PolyA tailed to allow subsequent insertion in TOPO vector. Subsequently, the PCR products were ligated into pCR2.1-TOPO vector using the TOPO TA Cloning kit (Thermo Fisher Scientific). Briefly, 4µl of polyA-tailed PCR product were mixed with 1µl of TOPO vector and 1µl of salt solution and the reaction was incubated for 30 min at RT allowing the ligation of the PCR product into the TOPO vector via recognition with the thymidine overhangs of the linearized vector.

Bacterial transformation and plasmid DNA extraction

One Shot® TOP10 Chemically competent *E. coli* (Thermo Fisher Scientific) were transformed using plasmid DNA and kept under ampicillin treatment (100µg/ml). Plasmid DNA from selected colonies was extracted using QIAprep Spin Miniprep Kit (Qiagen) and sequencing was performed at Eurofins Genomics. For preparation of the plasmids for transfection, the selected bacterial clones were allowed to grow in 250 ml of LB Broth with ampicillin overnight and plasmid DNA was extracted using HiSpeed Plasmid Maxi Kit (Qiagen).

Generation of LHA and RHA

The LHA was generated in one step (Fig. S1). The TOPO_HDR was amplified with the primer pair LHA_F/LHA_R with the GXL polymerase (Fig. S1A). The resulting PCR product was polyA tailed and cloned into a TOPO vector. After transformation, 5 independent colonies were screened via amplification with the M13 primers (Fig. S1B). Only the clones presenting a unique band at the expected size of 833bp were submitted to sequencing (LHA + sequence of TOPO between M13 primer binding sites = 663+170bp) with the M13 forward primer (Fig. S1C).

The RHA was generated in two steps (Fig. S1). The TOPO_HDR was amplified with the primer pair RHA_F/RHA_R with the GXL polymerase (Fig. S1A). The resulting PCR product was polyA tailed and cloned into a TOPO vector. After transformation, independent colonies were screened via amplification with the M13 primers (Fig. S1B). Only the clones presenting a unique band at the expected size of 1088bp were submitted to sequencing (LHA + sequence of TOPO between M13 primer binding sites = 918+170bp) with the M13 forward primer. This first step also allowed to silent-mutate the PAM sequence TGG>TCG (Fig. S1D, light blue). In the second step, we generated the TTAA motif and created a RHA to correct the mutation (harbouring AAC codon) or to insert

the mutation (harbouring AGC codon) via amplification of the selected TOPO_RHA (Clone_10) with different forward primers (RHAF_step2_N370 and RHAF_step2_N370S) (Fig. S1E). Subcloning into TOPO vector and sequencing with M13 primers allowed us to identify clones with correct sequence for correction of the mutation (Clone_4 = TOPO_RHA_N370) and for insertion of the mutation (Clone_11 = TOPO_RHA_N370S) (Fig. S1F and G).

Cell culture

The iPSC lines were maintained on Geltrex (Gibco) coated plates under E8 medium composed of DMEM/F12 (ThermoFisher) supplemented with 1% Insulin-Transferrin (Life/Tech), 1% Penicillin-Streptomycin (Life/Tech), 64µg/ml of ascorbic acid (Sigma-Aldrich), 20ng/ml of FGF-2 (Peprotech), 2ng/ml of TGF-β1 (Peprotech) and 100ng/ml Heparin (Sigma-Aldrich). To improve maintenance conditions, 10% of mTesR1 (Stem cell technologies) was added to the abovementioned media. Media was changed every day. Geltrex coating was performed by diluting concentrated matrix in ice-cold DMEM/F12 medium following manufacturer's instructions. The solution was allowed to coat for at least 1h at 37°C. Every 5 to 7 days iPSCs were split at a 1:5 to 1:10 ratio using 0.5 mM EDTA (Sigma-Aldrich). When iPSC were needed as single cells, culture were detached with Accutase (Sigma-Aldrich) and replated in media containing 10µM of ROCK inhibitor Y-27632 (Abcam). Cell cultures were maintained in a humidified incubator set at 37°C and 5% CO2.

Transfection of iPSCs

For transfection, $5x10^6$ living iPSC were collected with Accutase. Five transfection were realized with 1.10^6 cells per transfection. The Human Stem Cell Nucleofector Kit 1 (Lonza) was used and cells were electroporated using the B16 program from Amaxa nucleofector II (Lonza). After electroporation, cells were allowed to stay in the cuvette at 37°C for 5 min as we observed a better survival after completing this step. Then all eletroporated cells were platted in two Geltrex pre-coated 1 well plate NuncTM OmniTrayTM (Thermo Fisher Scientific) in media supplemented with ROCK inhibitor.

iPSC-Colony PCR :

For PCR-based screening, colonies were lysed in colony-PCR lysis buffer (105 mM KCl, 14 mM Tris-HCl, pH 8.3, 2.5 mM MgCl2, 0.3 mg ml − 1 gelatin, 0.45% (vol/vol) NP-40 and 0.45% (vol/vol) Tween 20) (Papapetrou et al. 2011) supplemented with 10% proteinase K (Qiagen). Samples were heated at 55°C for 1hr followed by 15 min at 95°C to inactivate the proteinase K. 3µl of the lysate was used for PCR amplification with the Gotaq G2 Flexi DNA Polymerase (Promega).

Sequencing:

For sequencing, DNA was extracted with the DNeasy Blood & Tissue kit (Qiagen) and amplified with the high-fidelity KOD Hot Start polymerase (Merck Millipore). For GBA and GBAP1 amplification, 5% DMSO was added to the PCR mix. For sequencing of GBA, amplification primer forward VKI_Pr1_GBA_F (5'-ggaggctaatgtgggaggat-3') combined to reverse primer SEQPRA_Pr1_GBA_R (5'-ACCACCTAGAGGGGAAAGTG-3') and sequencing primer VKI_SEQ_GBA_F (5'-CACAGGGCTGACCTACCCAC-3') were used. For sequencing of GBAP1, only the forward primer was different: NKI_GBAP1_F (5'-TCAAGATGAGCCTGGGAAAC-3'). Sequencing was performed by Eurofins Genomics.

Immunofluorescence

For characterization of pluripotency, iPSC were platted on Geltrex-coated coverslips and fixed with 4% PFA (Merck Millipore). After permeabilization, iPSC were incubated with first antibody against Nanog (abcam – ab21624 – 1:250), Oct4 (Santa Cruz – sc-5279 – 1:250) or Sox2 (Santa Cruz – sc-17320 – 1:250) overnight at 4°C. Fluorescent conjugated antibodies were then incubated for 3 hours at room temperature (Invitrogen – A11036, A11029 and A21447). Coverslips were stained with Hoechst (Invitrogen) for 15 min and mounted on slides. Image were acquired on a confocal spinning disc microscope (Axiovert 2000), with a Hamamatsu camera C11440 with the x25 objective in water-based immersion solution (Imersol).

GCase activity assay

GCase activity assay was performed on 500 000 cells. iPSCs were detached with Accutase, centrifuged at 300g for 3 min and resuspended in 50µl of GCase activity assay buffer (0.25% (v/v) Triton X-100 (Sigma-Aldrich), 0.25% (w/v) Taurocholic acid (Sigma-Aldrich), 1 mM EDTA, in citrate/phosphate buffer, pH 5.4). Resuspended cells were frozen/thawed twice and incubated on ice for 30 min. Then they were centrifuged at 20 000g for 20 minutes and 10µl of supernatant was used to assess GCase activity in triplicate in 3 wells of a black 96wp for fluorescence. For this, 40 µl of 1 mM 4-Methylumbelliferyl □-glucophyranoside (4-MU, Sigma-Aldrich) with 1% BSA was added to each replicate and the mix was left at 37°C for 40 minutes. To stop the reaction, an equivolume (50µl) of 1M Glycine was added to each well. Fluorescent reading (ex=355nm, em=460, 0.1 second) was performed on an Infinite® M200 PRO (Tecan) using Magelan software. Total protein amount of each sample was quantify by Pierce BCA assay (Thermo Fisher Scientific). GCase activity was expressed in fluorescence intensity normalized to total protein level.

Western blotting

Cells were harvested with Accutase (Sigma-Aldrich) and lysed in an adequate volume of Triton X-100 based lysis buffer. This cell lysate was submitted to total protein quantification using Bradford (Bio-rad) and 20µg of total protein was used for Western blotting. 6xLämli buffer was added to protein lysate allowing denaturation at 96°C for 7 min. After SDS-PAGE, the gels were transferred to nitrocellulose membrane via dry transfer (iBlot 2 – Thermo Fisher Scientific). The membranes were blocked in TBS containing 5% milk for 1 hour at room temperature and incubated overnight at 4°C with first antibody against GCase (Abnova - H00002629-M01, 1:3000) or b-actin (Cell signalling - 3700S, 1:20 000) diluted in TBS containing 5% milk. The next day, membranes were washed and incubated with secondary antibody anti-mouse HRP-conjugated (Invitrogen) for 1 hour at room temperature. Membranes were revealed using Amersham ECL solution (Sigma-Alrdich) on LICOR Odyssey Fc. Densitometry of bands was obtained by using the Image Studio software and each band of protein of interest was normalized to b-actin.

Statistical analysis.

Each experiment was performed on iPSCs from at least five independent passages. All data were pooled and analysed in Prism GraphPad 8.1.0. The difference between isogenic lines was assessed by two-tailed paired t-test, with a significance level of 5%.
