# Supplementary material for: Quality Control Strategy for CRISPR-Cas9-Based Gene Editing Complicated by a Pseudogene
Source: Front Genet. 2020 Jan 8;10:1297. doi: 10.3389/fgene.2019.01297 (PMC6961559; doi:10.3389/fgene.2019.01297)
Supplement: Supplementary file 2 [file Table_1.docx]

**Supplementary Table 1:** Primers used in this study

| Primer names | Sequence (5’-3’) | Function | Amplicon size | Tm |
| --- | --- | --- | --- | --- |
| *Extraction of region of interest from genomic DNA* | | | | |
| SEQPRA_Pr1_GBA_F | TGTGTGCAAGGTCCAGGATCAG | Amplification homology arms GBA | 1682bp | 52°C |
| SEQPRA_Pr1_GBA_R | ACCACCTAGAGGGGAAAGTG |  |  |  |
| SEQPRA_Pr1_GBAP1_F | CAGGGTCCAGGGTGACTTGTT | Amplification of GBAP1 | 1621bp | 52°C |
| SEQPRA_Pr1_GBA_R | ACCACCTAGAGGGGAAAGTG |  |  |  |
| *Construction of donor vector* | | | | |
| LHA_F | AAGCTTGGATCCCCTAGGTTTGCAGGTACTGACAGACCCAG | Amplification LHA from TOPO | 663bp | 60°C |
| LHA_R | CAGACTATCTTTCTAGGGTTAAGGACAAAGGCAAAGAGAC |  |  |  |
| RHA_F_step1 | CTCCTGTACCATGTCGTCGGCTGGACCGAC | Amplification RHA from TOPO  (2 steps) | 918bp | 60°C |
| RHA_R | GCATACGCGTATACTAGGTTGTCCCTTTAATGCCCAGGCTGAG |  |  |  |
| RHA_F_step2_N370 | ATGATTATCTTTCTAGGGTTAACCTAGAACCTCCTGTACCATGTCG |  |  |  |
| RHA_F_step2_N370S | ATGATTATCTTTCTAGGGTTAACCTAGAGCCTCCTGTACCATGTCG |  |  |  |
| *Construction of sgRNA Cas9 vector* | | | | |
| gRNA_on-N370S_F | CACCGCTAGAGCCTCCTGTACCATG | Creation of sgRNA vector for correction of N370S |  |  |
| gRNA_on-N370S_R | AAACCATGGTACAGGAGGCTCTAGC |  |  |  |
| gRNA_on-N370_F | CACCGCTAGAACCTCCTGTACCATG | Creation of sgRNA vector for  Insertion of N370S |  |  |
| gRNA_on-N370_R | AAACCATGGTACAGGAGGTTCTAGC |  |  |  |
| *Screening primers to validate KI* | | | | |
| VKI_GBA_F | GGAGGCTAATGTGGGAGGAT | Validation correct editing of GBA | 1000bp | 58°C |
| VKI_GBA_R | AGATGTCCTAAATGCACAGCG |  |  |  |
| NKI_GBAP1_F | TCAAGATGAGCCTGGGAAAC | Validation GBAP1 unaltered | 1027bp | 58°C |
| NKI_GBAP1_R | GTCTACAATGATGGGTTCCAG |  |  |  |
| VKI_GBA_F | GGAGGCTAATGTGGGAGGAT | Evaluation non editing of GBA | 952nt | 58°C |
| NKI_GBA_R | GGACTGTCGACAAAGTTACGC |  |  |  |
| VKI_LHA_F | GCTGCCTATCAGAAGGTGGTG | Random integration LHA | 1446bp | 60°C |
| VKI_Pr1_R | AGATGTCCTAAATGCACAGCG |  |  |  |
| VKI_RHA_F | CGATATACAGACCGATAAAACACATGC | Random integration RHA | 1666bp | 60°C |
| VKI_RHA_R | GCAGCCACTGGTAACAGGAT |  |  |  |
| VKI_SEQ_GBA_F | CACAGGGCTGACCTACCCAC | Final sequencing GBA |  |  |
